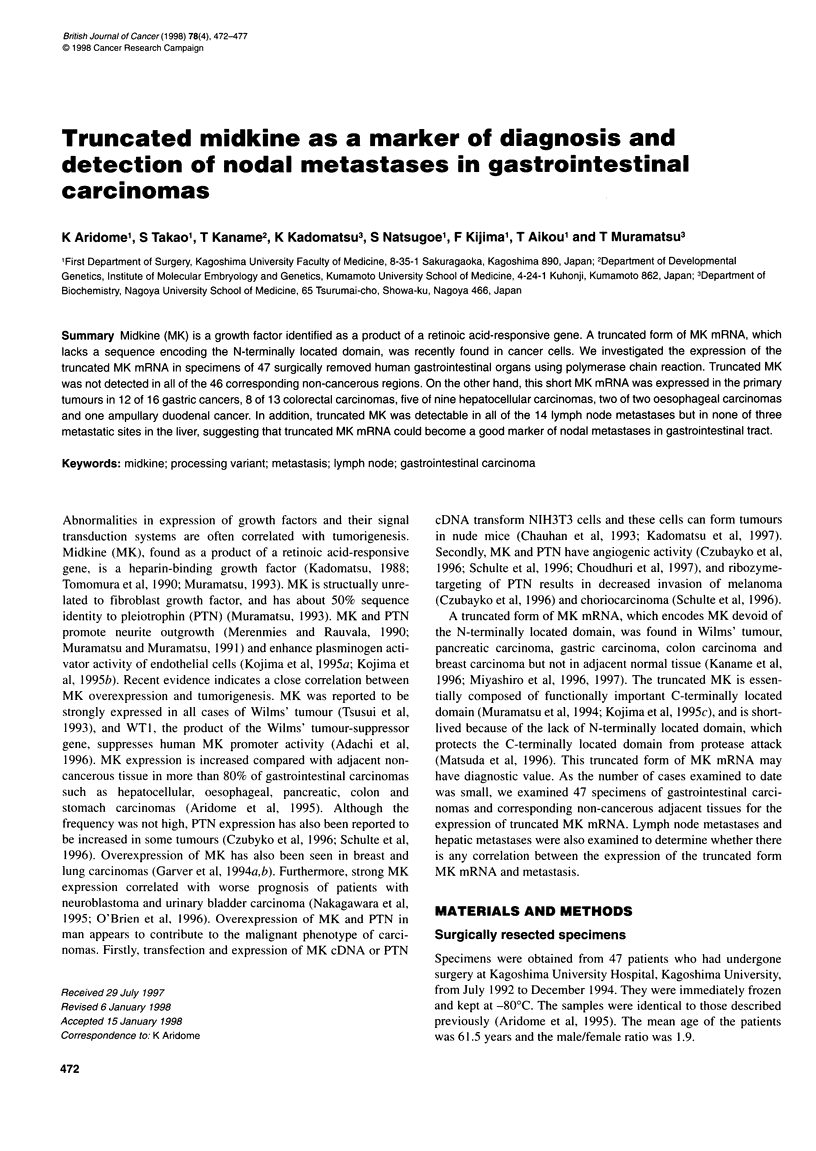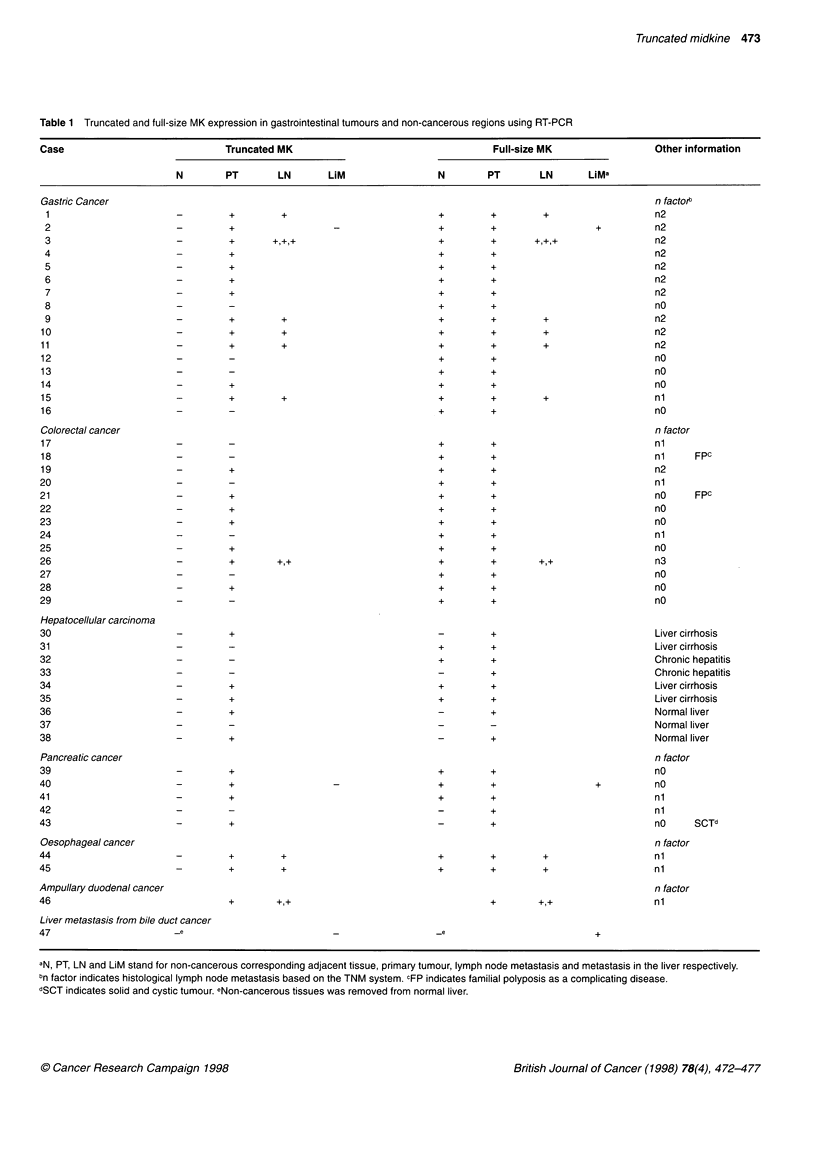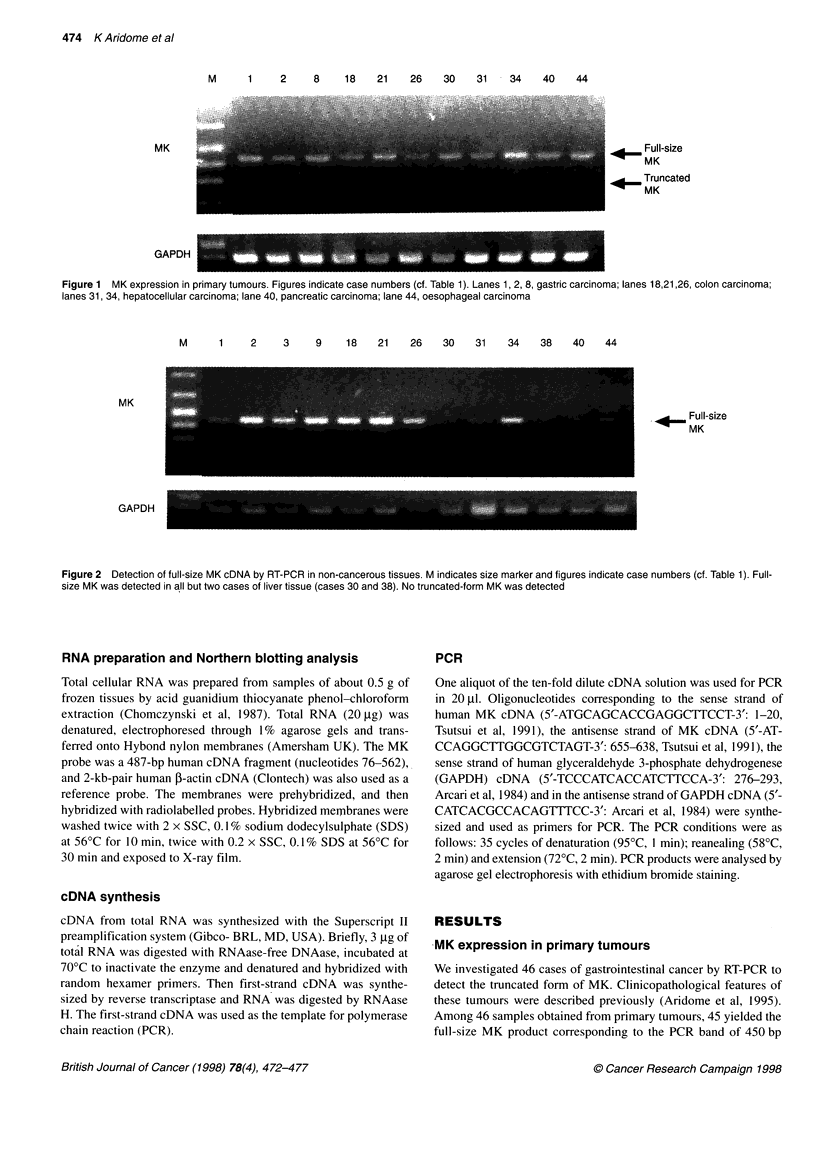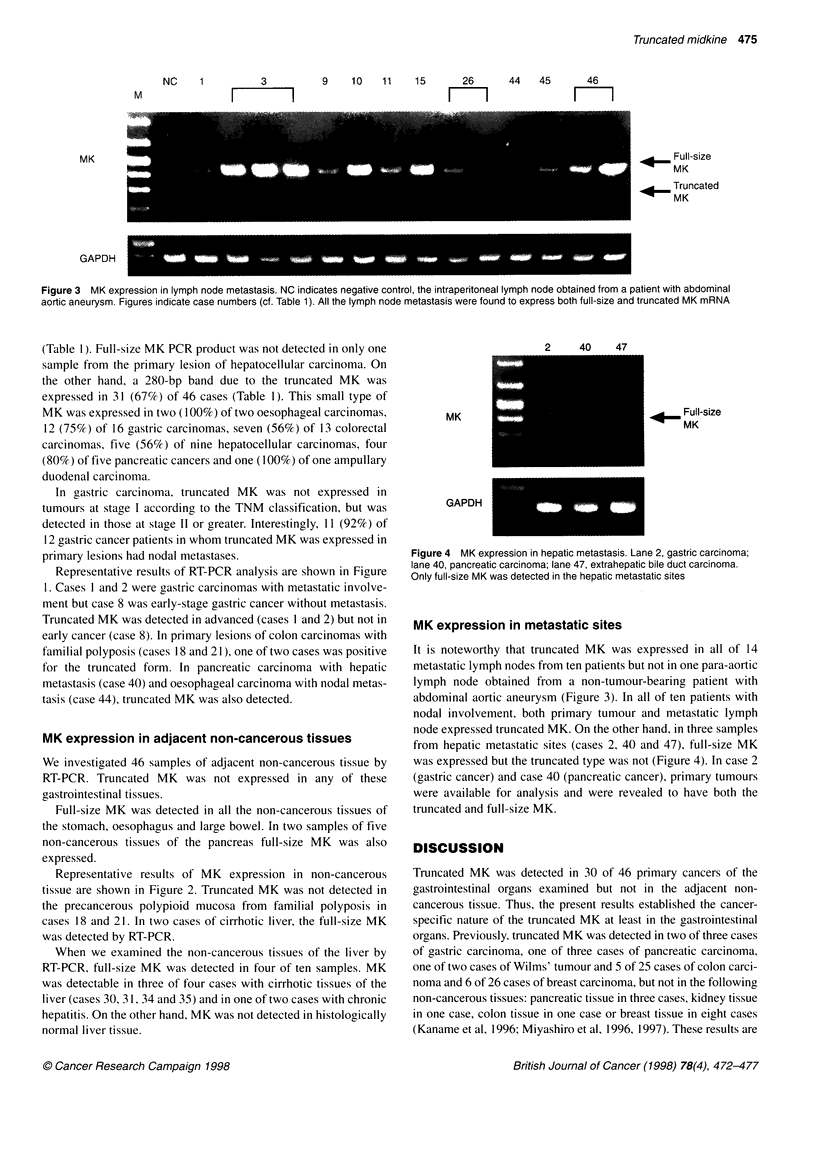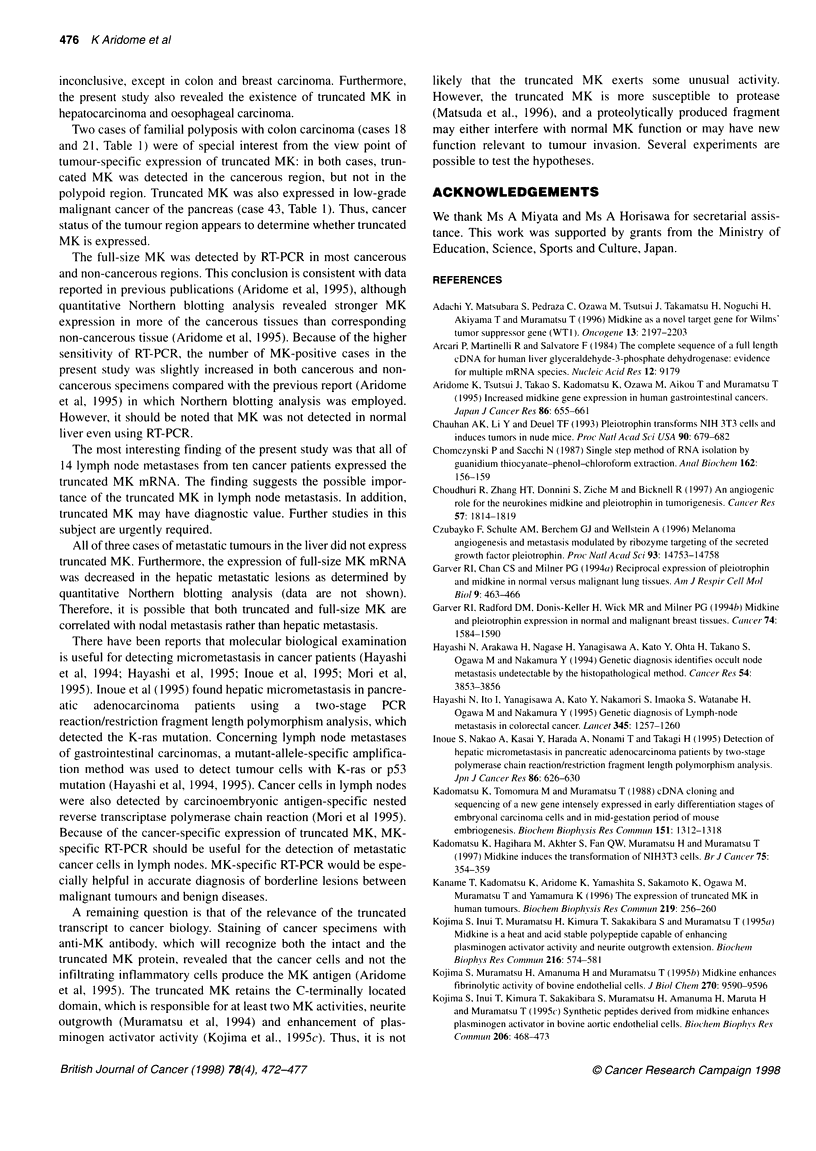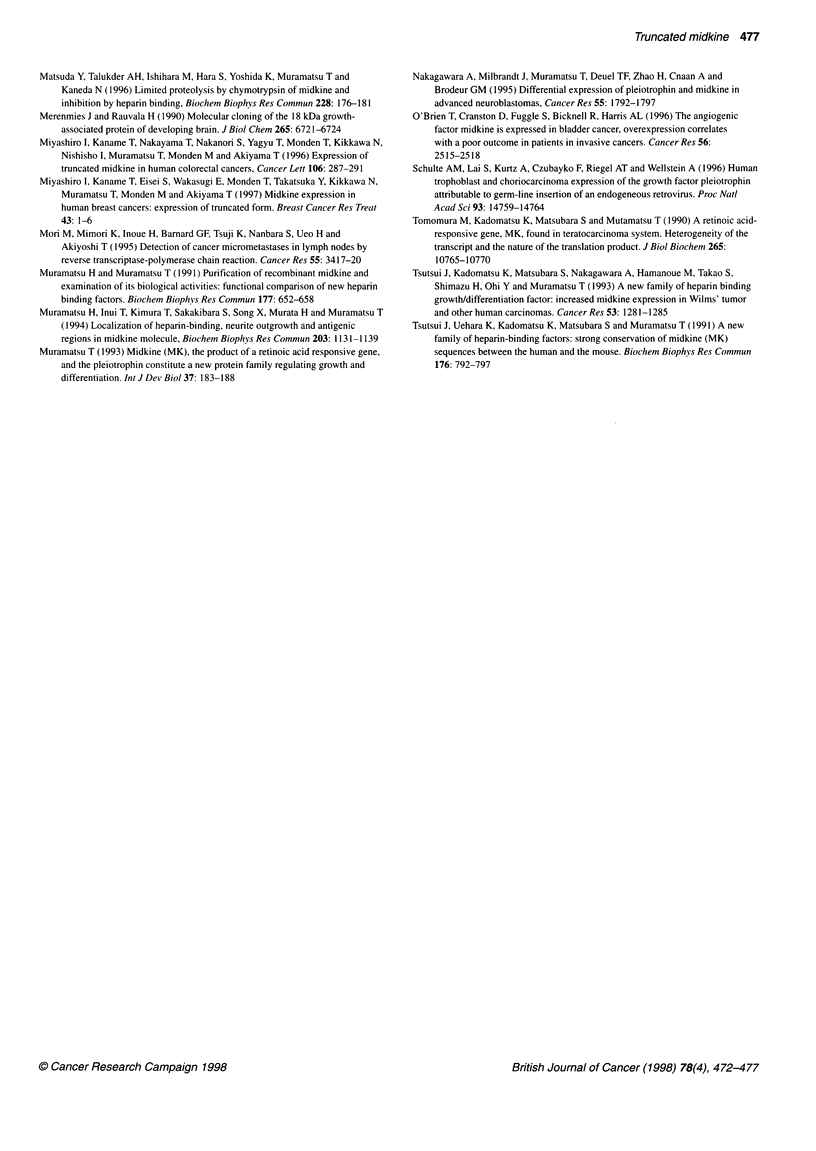# Truncated midkine as a marker of diagnosis and detection of nodal metastases in gastrointestinal carcinomas.

**DOI:** 10.1038/bjc.1998.517

**Published:** 1998-08

**Authors:** K. Aridome, S. Takao, T. Kaname, K. Kadomatsu, S. Natsugoe, F. Kijima, T. Aikou, T. Muramatsu

**Affiliations:** First Department of Surgery, Kagoshima University Faculty of Medicine, Japan.

## Abstract

**Images:**


British Journal of Cancer (1998) 78(4), 472–477
© 1998 Cancer Research Campaign

### Truncated midkine as a marker of diagnosis and detection of nodal metastases in gastrointestinal carcinomas

**K Aridome[1], S Takao[1], T Kaname[2], K Kadomatsu[3], S Natsugoe[1], F Kijima[1], T Aikou[1] and T Muramatsu[3]**

[1]First Department of Surgery, Kagoshima University Faculty of Medicine, 8-35-1 Sakuragaoka, Kagoshima 890, Japan; [2]Department of Developmental Genetics, Institute of Molecular Embryology and Genetics, Kumamoto University School of Medicine, 4-24-1 Kuhonji, Kumamoto 862, Japan; [3]Department of Biochemistry, Nagoya University School of Medicine, 65 Tsurumai-cho, Showa-ku, Nagoya 466, Japan

**Summary** Midkine (MK) is a growth factor identified as a product of a retinoic acid-responsive gene. A truncated form of MK mRNA, which lacks a sequence encoding the N-terminally located domain, was recently found in cancer cells. We investigated the expression of the truncated MK mRNA in specimens of 47 surgically removed human gastrointestinal organs using polymerase chain reaction. Truncated MK was not detected in all of the 46 corresponding non-cancerous regions. On the other hand, this short MK mRNA was expressed in the primary tumours in 12 of 16 gastric cancers, 8 of 13 colorectal carcinomas, five of nine hepatocellular carcinomas, two of two oesophageal carcinomas and one ampullary duodenal cancer. In addition, truncated MK was detectable in all of the 14 lymph node metastases but in none of three metastatic sites in the liver, suggesting that truncated MK mRNA could become a good marker of nodal metastases in gastrointestinal tract.

**Keywords:** midkine; processing variant; metastasis; lymph node; gastrointestinal carcinoma

Abnormalities in expression of growth factors and their signal transduction systems are often correlated with tumorigenesis. Midkine (MK), found as a product of a retinoic acid-responsive gene, is a heparin-binding growth factor (Kadomatsu, 1988; Tomomura et al, 1990; Muramatsu, 1993). MK is structually unrelated to fibroblast growth factor, and has about 50% sequence identity to pleiotrophin (PTN) (Muramatsu, 1993). MK and PTN promote neurite outgrowth (Merenmies and Rauvala, 1990; Muramatsu and Muramatsu, 1991) and enhance plasminogen activator activity of endothelial cells (Kojima et al, 1995*a*; Kojima et al, 1995*b*). Recent evidence indicates a close correlation between MK overexpression and tumorigenesis. MK was reported to be strongly expressed in all cases of Wilms' tumour (Tsusui et al, 1993), and WT1, the product of the Wilms' tumour-suppressor gene, suppresses human MK promoter activity (Adachi et al, 1996). MK expression is increased compared with adjacent non-cancerous tissue in more than 80% of gastrointestinal carcinomas such as hepatocellular, oesophageal, pancreatic, colon and stomach carcinomas (Aridome et al, 1995). Although the frequency was not high, PTN expression has also been reported to be increased in some tumours (Czubyko et al, 1996; Schulte et al, 1996). Overexpression of MK has also been seen in breast and lung carcinomas (Garver et al, 1994*a,b*). Furthermore, strong MK expression correlated with worse prognosis of patients with neuroblastoma and urinary bladder carcinoma (Nakagawara et al, 1995; O'Brien et al, 1996). Overexpression of MK and PTN in man appears to contribute to the malignant phenotype of carcinomas. Firstly, transfection and expression of MK cDNA or PTN cDNA transform NIH3T3 cells and these cells can form tumours in nude mice (Chauhan et al, 1993; Kadomatsu et al, 1997). Secondly, MK and PTN have angiogenic activity (Czubayko et al, 1996; Schulte et al, 1996; Choudhuri et al, 1997), and ribozyme-targeting of PTN results in decreased invasion of melanoma (Czubayko et al, 1996) and choriocarcinoma (Schulte et al, 1996).

A truncated form of MK mRNA, which encodes MK devoid of the N-terminally located domain, was found in Wilms' tumour, pancreatic carcinoma, gastric carcinoma, colon carcinoma and breast carcinoma but not in adjacent normal tissue (Kaname et al, 1996; Miyashiro et al, 1996, 1997). The truncated MK is essentially composed of functionally important C-terminally located domain (Muramatsu et al, 1994; Kojima et al, 1995*c*), and is short-lived because of the lack of N-terminally located domain, which protects the C-terminally located domain from protease attack (Matsuda et al, 1996). This truncated form of MK mRNA may have diagnostic value. As the number of cases examined to date was small, we examined 47 specimens of gastrointestinal carcinomas and corresponding non-cancerous adjacent tissues for the expression of truncated MK mRNA. Lymph node metastases and hepatic metastases were also examined to determine whether there is any correlation between the expression of the truncated form MK mRNA and metastasis.

### MATERIALS AND METHODS

### Surgically resected specimens

Specimens were obtained from 47 patients who had undergone surgery at Kagoshima University Hospital, Kagoshima University, from July 1992 to December 1994. They were immediately frozen and kept at –80°C. The samples were identical to those described previously (Aridome et al, 1995). The mean age of the patients was 61.5 years and the male/female ratio was 1.9.

*Received 29 July 1997*
*Revised 6 January 1998*
*Accepted 15 January 1998*
*Correspondence to:* K Aridome

**Table 1** Truncated and full-size MK expression in gastrointestinal tumours and non-cancerous regions using RT-PCR

| Case | Truncated MK | | | | Full-size MK | | | | Other information |
|---|---|---|---|---|---|---|---|---|---|
| | N | PT | LN | LiM | N | PT | LN | LiM[a] | |
| *Gastric Cancer* | | | | | | | | | *n factor*[b] |
| 1 | – | + | + | | + | + | + | | n2 |
| 2 | – | + | | – | + | + | | + | n2 |
| 3 | – | + | +,+,+ | | + | + | +,+,+ | | n2 |
| 4 | – | + | | | + | + | | | n2 |
| 5 | – | + | | | + | + | | | n2 |
| 6 | – | + | | | + | + | | | n2 |
| 7 | – | + | | | + | + | | | n2 |
| 8 | – | – | | | + | + | | | n0 |
| 9 | – | + | + | | + | + | + | | n2 |
| 10 | – | + | + | | + | + | + | | n2 |
| 11 | – | + | + | | + | + | + | | n2 |
| 12 | – | – | | | + | + | | | n0 |
| 13 | – | – | | | + | + | | | n0 |
| 14 | – | + | | | + | + | | | n0 |
| 15 | – | + | + | | + | + | + | | n1 |
| 16 | – | – | | | + | + | | | n0 |
| *Colorectal cancer* | | | | | | | | | *n factor* |
| 17 | – | – | | | + | + | | | n1 |
| 18 | – | – | | | + | + | | | n1 FP[c] |
| 19 | – | + | | | + | + | | | n2 |
| 20 | – | – | | | + | + | | | n1 |
| 21 | – | + | | | + | + | | | n0 FP[c] |
| 22 | – | + | | | + | + | | | n0 |
| 23 | – | + | | | + | + | | | n0 |
| 24 | – | – | | | + | + | | | n1 |
| 25 | – | + | | | + | + | | | n0 |
| 26 | – | + | +,+ | | + | + | +,+ | | n3 |
| 27 | – | – | | | + | + | | | n0 |
| 28 | – | + | | | + | + | | | n0 |
| 29 | – | – | | | + | + | | | n0 |
| *Hepatocellular carcinoma* | | | | | | | | | |
| 30 | – | + | | | – | + | | | Liver cirrhosis |
| 31 | – | – | | | + | + | | | Liver cirrhosis |
| 32 | – | – | | | + | + | | | Chronic hepatitis |
| 33 | – | – | | | – | + | | | Chronic hepatitis |
| 34 | – | + | | | + | + | | | Liver cirrhosis |
| 35 | – | + | | | + | + | | | Liver cirrhosis |
| 36 | – | + | | | – | + | | | Normal liver |
| 37 | – | – | | | – | – | | | Normal liver |
| 38 | – | + | | | – | + | | | Normal liver |
| *Pancreatic cancer* | | | | | | | | | *n factor* |
| 39 | – | + | | | + | + | | | n0 |
| 40 | – | + | | – | + | + | | + | n0 |
| 41 | – | + | | | + | + | | | n1 |
| 42 | – | – | | | – | + | | | n1 |
| 43 | – | + | | | – | + | | | n0 SCT[d] |
| *Oesophageal cancer* | | | | | | | | | *n factor* |
| 44 | – | + | + | | + | + | + | | n1 |
| 45 | – | + | + | | + | + | + | | n1 |
| *Ampullary duodenal cancer* | | | | | | | | | *n factor* |
| 46 | | + | +,+ | | | + | +,+ | | n1 |
| *Liver metastasis from bile duct cancer* | | | | | | | | | |
| 47 | –[e] | | | – | –[e] | | | + | |

[a]N, PT, LN and LiM stand for non-cancerous corresponding adjacent tissue, primary tumour, lymph node metastasis and metastasis in the liver respectively. [b]n factor indicates histological lymph node metastasis based on the TNM system. [c]FP indicates familial polyposis as a complicating disease. [d]SCT indicates solid and cystic tumour. [e]Non-cancerous tissues was removed from normal liver.

*© Cancer Research Campaign 1998*

**Figure 1** MK expression in primary tumours. Figures indicate case numbers (cf. Table 1). Lanes 1, 2, 8, gastric carcinoma; lanes 18,21,26, colon carcinoma; lanes 31, 34, hepatocellular carcinoma; lane 40, pancreatic carcinoma; lane 44, oesophageal carcinoma

**Figure 2** Detection of full-size MK cDNA by RT-PCR in non-cancerous tissues. M indicates size marker and figures indicate case numbers (cf. Table 1). Full-size MK was detected in all but two cases of liver tissue (cases 30 and 38). No truncated-form MK was detected

### RNA preparation and Northern blotting analysis

Total cellular RNA was prepared from samples of about 0.5 g of frozen tissues by acid guanidium thiocyanate phenol–chloroform extraction (Chomczynski et al, 1987). Total RNA (20 μg) was denatured, electrophoresed through 1% agarose gels and transferred onto Hybond nylon membranes (Amersham UK). The MK probe was a 487-bp human cDNA fragment (nucleotides 76–562), and 2-kb-pair human β-actin cDNA (Clontech) was also used as a reference probe. The membranes were prehybridized, and then hybridized with radiolabelled probes. Hybridized membranes were washed twice with 2 × SSC, 0.1% sodium dodecylsulphate (SDS) at 56°C for 10 min, twice with 0.2 × SSC, 0.1% SDS at 56°C for 30 min and exposed to X-ray film.

### cDNA synthesis cDNA from total RNA was synthesized with the Superscript II preamplification system (Gibco- BRL, MD, USA). Briefly, 3 μg of total RNA was digested with RNAase-free DNAase, incubated at 70°C to inactivate the enzyme and denatured and hybridized with random hexamer primers. Then first-strand cDNA was synthesized by reverse transcriptase and RNA was digested by RNAase H. The first-strand cDNA was used as the template for polymerase chain reaction (PCR).

### PCR

One aliquot of the ten-fold dilute cDNA solution was used for PCR in 20 μl. Oligonucleotides corresponding to the sense strand of human MK cDNA (5′-ATGCAGCACCGAGGCTTCCT-3′: 1–20, Tsutsui et al, 1991), the antisense strand of MK cDNA (5′-ATCCAGGCTTGGCGTCTAGT-3′: 655–638, Tsutsui et al, 1991), the sense strand of human glyceraldehyde 3-phosphate dehydrogenese (GAPDH) cDNA (5′-TCCCATCACCATCTTCCA-3′: 276–293, Arcari et al, 1984) and in the antisense strand of GAPDH cDNA (5′-CATCACGCCACAGTTTCC-3′: Arcari et al, 1984) were synthesized and used as primers for PCR. The PCR conditions were as follows: 35 cycles of denaturation (95°C, 1 min); reanealing (58°C, 2 min) and extension (72°C, 2 min). PCR products were analysed by agarose gel electrophoresis with ethidium bromide staining.

### RESULTS

### MK expression in primary tumours

We investigated 46 cases of gastrointestinal cancer by RT-PCR to detect the truncated form of MK. Clinicopathological features of these tumours were described previously (Aridome et al, 1995). Among 46 samples obtained from primary tumours, 45 yielded the full-size MK product corresponding to the PCR band of 450 bp

 © Cancer Research Campaign 1998

**Figure 3** MK expression in lymph node metastasis. NC indicates negative control, the intraperitoneal lymph node obtained from a patient with abdominal aortic aneurysm. Figures indicate case numbers (cf. Table 1). All the lymph node metastasis were found to express both full-size and truncated MK mRNA

(Table 1). Full-size MK PCR product was not detected in only one sample from the primary lesion of hepatocellular carcinoma. On the other hand, a 280-bp band due to the truncated MK was expressed in 31 (67%) of 46 cases (Table 1). This small type of MK was expressed in two (100%) of two oesophageal carcinomas, 12 (75%) of 16 gastric carcinomas, seven (56%) of 13 colorectal carcinomas, five (56%) of nine hepatocellular carcinomas, four (80%) of five pancreatic cancers and one (100%) of one ampullary duodenal carcinoma.

In gastric carcinoma, truncated MK was not expressed in tumours at stage I according to the TNM classification, but was detected in those at stage II or greater. Interestingly, 11 (92%) of 12 gastric cancer patients in whom truncated MK was expressed in primary lesions had nodal metastases.

Representative results of RT-PCR analysis are shown in Figure 1. Cases 1 and 2 were gastric carcinomas with metastatic involvement but case 8 was early-stage gastric cancer without metastasis. Truncated MK was detected in advanced (cases 1 and 2) but not in early cancer (case 8). In primary lesions of colon carcinomas with familial polyposis (cases 18 and 21), one of two cases was positive for the truncated form. In pancreatic carcinoma with hepatic metastasis (case 40) and oesophageal carcinoma with nodal metastasis (case 44), truncated MK was also detected.

### MK expression in adjacent non-cancerous tissues

We investigated 46 samples of adjacent non-cancerous tissue by RT-PCR. Truncated MK was not expressed in any of these gastrointestinal tissues.

Full-size MK was detected in all the non-cancerous tissues of the stomach, oesophagus and large bowel. In two samples of five non-cancerous tissues of the pancreas full-size MK was also expressed.

Representative results of MK expression in non-cancerous tissue are shown in Figure 2. Truncated MK was not detected in the precancerous polypioid mucosa from familial polyposis in cases 18 and 21. In two cases of cirrhotic liver, the full-size MK was detected by RT-PCR.

When we examined the non-cancerous tissues of the liver by RT-PCR, full-size MK was detected in four of ten samples. MK was detectable in three of four cases with cirrhotic tissues of the liver (cases 30, 31, 34 and 35) and in one of two cases with chronic hepatitis. On the other hand, MK was not detected in histologically normal liver tissue.

**Figure 4** MK expression in hepatic metastasis. Lane 2, gastric carcinoma; lane 40, pancreatic carcinoma; lane 47, extrahepatic bile duct carcinoma. Only full-size MK was detected in the hepatic metastatic sites

### MK expression in metastatic sites

It is noteworthy that truncated MK was expressed in all of 14 metastatic lymph nodes from ten patients but not in one para-aortic lymph node obtained from a non-tumour-bearing patient with abdominal aortic aneurysm (Figure 3). In all of ten patients with nodal involvement, both primary tumour and metastatic lymph node expressed truncated MK. On the other hand, in three samples from hepatic metastatic sites (cases 2, 40 and 47), full-size MK was expressed but the truncated type was not (Figure 4). In case 2 (gastric cancer) and case 40 (pancreatic cancer), primary tumours were available for analysis and were revealed to have both the truncated and full-size MK.

### DISCUSSION

Truncated MK was detected in 30 of 46 primary cancers of the gastrointestinal organs examined but not in the adjacent non-cancerous tissue. Thus, the present results established the cancer-specific nature of the truncated MK at least in the gastrointestinal organs. Previously, truncated MK was detected in two of three cases of gastric carcinoma, one of three cases of pancreatic carcinoma, one of two cases of Wilms' tumour and 5 of 25 cases of colon carcinoma and 6 of 26 cases of breast carcinoma, but not in the following non-cancerous tissues: pancreatic tissue in three cases, kidney tissue in one case, colon tissue in one case or breast tissue in eight cases (Kaname et al, 1996; Miyashiro et al, 1996, 1997). These results are

© Cancer Research Campaign 1998

inconclusive, except in colon and breast carcinoma. Furthermore, the present study also revealed the existence of truncated MK in hepatocarcinoma and oesophageal carcinoma.

Two cases of familial polyposis with colon carcinoma (cases 18 and 21, Table 1) were of special interest from the view point of tumour-specific expression of truncated MK: in both cases, truncated MK was detected in the cancerous region, but not in the polypoid region. Truncated MK was also expressed in low-grade malignant cancer of the pancreas (case 43, Table 1). Thus, cancer status of the tumour region appears to determine whether truncated MK is expressed.

The full-size MK was detected by RT-PCR in most cancerous and non-cancerous regions. This conclusion is consistent with data reported in previous publications (Aridome et al, 1995), although quantitative Northern blotting analysis revealed stronger MK expression in more of the cancerous tissues than corresponding non-cancerous tissue (Aridome et al, 1995). Because of the higher sensitivity of RT-PCR, the number of MK-positive cases in the present study was slightly increased in both cancerous and non-cancerous specimens compared with the previous report (Aridome et al, 1995) in which Northern blotting analysis was employed. However, it should be noted that MK was not detected in normal liver even using RT-PCR.

The most interesting finding of the present study was that all of 14 lymph node metastases from ten cancer patients expressed the truncated MK mRNA. The finding suggests the possible importance of the truncated MK in lymph node metastasis. In addition, truncated MK may have diagnostic value. Further studies in this subject are urgently required.

All of three cases of metastatic tumours in the liver did not express truncated MK. Furthermore, the expression of full-size MK mRNA was decreased in the hepatic metastatic lesions as determined by quantitative Northern blotting analysis (data are not shown). Therefore, it is possible that both truncated and full-size MK are correlated with nodal metastasis rather than hepatic metastasis.

There have been reports that molecular biological examination is useful for detecting micrometastasis in cancer patients (Hayashi et al, 1994; Hayashi et al, 1995; Inoue et al, 1995; Mori et al, 1995). Inoue et al (1995) found hepatic micrometastasis in pancreatic adenocarcinoma patients using a two-stage PCR reaction/restriction fragment length polymorphism analysis, which detected the K-ras mutation. Concerning lymph node metastases of gastrointestinal carcinomas, a mutant-allele-specific amplification method was used to detect tumour cells with K-ras or p53 mutation (Hayashi et al, 1994, 1995). Cancer cells in lymph nodes were also detected by carcinoembryonic antigen-specific nested reverse transcriptase polymerase chain reaction (Mori et al 1995). Because of the cancer-specific expression of truncated MK, MK-specific RT-PCR should be useful for the detection of metastatic cancer cells in lymph nodes. MK-specific RT-PCR would be especially helpful in accurate diagnosis of borderline lesions between malignant tumours and benign diseases.

A remaining question is that of the relevance of the truncated transcript to cancer biology. Staining of cancer specimens with anti-MK antibody, which will recognize both the intact and the truncated MK protein, revealed that the cancer cells and not the infiltrating inflammatory cells produce the MK antigen (Aridome et al, 1995). The truncated MK retains the C-terminally located domain, which is responsible for at least two MK activities, neurite outgrowth (Muramatsu et al, 1994) and enhancement of plasminogen activator activity (Kojima et al., 1995*c*). Thus, it is not likely that the truncated MK exerts some unusual activity. However, the truncated MK is more susceptible to protease (Matsuda et al., 1996), and a proteolytically produced fragment may either interfere with normal MK function or may have new function relevant to tumour invasion. Several experiments are possible to test the hypotheses.

### ACKNOWLEDGEMENTS

We thank Ms A Miyata and Ms A Horisawa for secretarial assistance. This work was supported by grants from the Ministry of Education, Science, Sports and Culture, Japan.

### REFERENCES

Adachi Y, Matsubara S, Pedraza C, Ozawa M, Tsutsui J, Takamatsu H, Noguchi H, Akiyama T and Muramatsu T (1996) Midkine as a novel target gene for Wilms' tumor suppressor gene (WT1). *Oncogene* **13**: 2197–2203

Arcari P, Martinelli R and Salvatore F (1984) The complete sequence of a full length cDNA for human liver glyceraldehyde-3-phosphate dehydrogenase: evidence for multiple mRNA species. *Nucleic Acid Res* **12**: 9179

Aridome K, Tsutsui J, Takao S, Kadomatsu K, Ozawa M, Aikou T and Muramatsu T (1995) Increased midkine gene expression in human gastrointestinal cancers. *Japan J Cancer Res* **86**: 655–661

Chauhan AK, Li Y and Deuel TF (1993) Pleiotrophin transforms NIH 3T3 cells and induces tumors in nude mice. *Proc Natl Acad Sci USA* **90**: 679–682

Chomczynski P and Sacchi N (1987) Single step method of RNA isolation by guanidium thiocyanate–phenol–chloroform extraction. *Anal Biochem* **162**: 156–159

Choudhuri R, Zhang HT, Donnini S, Ziche M and Bicknell R (1997) An angiogenic role for the neurokines midkine and pleiotrophin in tumorigenesis. *Cancer Res* **57**: 1814–1819

Czubayko F, Schulte AM, Berchem GJ and Wellstein A (1996) Melanoma angiogenesis and metastasis modulated by ribozyme targeting of the secreted growth factor pleiotrophin. *Proc Natl Acad Sci* **93**: 14753–14758

Garver RI, Chan CS and Milner PG (1994*a*) Reciprocal expression of pleiotrophin and midkine in normal versus malignant lung tissues. *Am J Respir Cell Mol Biol* **9**: 463–466

Garver RI, Radford DM, Donis-Keller H, Wick MR and Milner PG (1994*b*) Midkine and pleiotrophin expression in normal and malignant breast tissues. *Cancer* **74**: 1584–1590

Hayashi N, Arakawa H, Nagase H, Yanagisawa A, Kato Y, Ohta H, Takano S, Ogawa M and Nakamura Y (1994) Genetic diagnosis identifies occult node metastasis undetectable by the histopathological method. *Cancer Res* **54**: 3853–3856

Hayashi N, Ito I, Yanagisawa A, Kato Y, Nakamori S, Imaoka S, Watanabe H, Ogawa M and Nakamura Y (1995) Genetic diagnosis of Lymph-node metastasis in colorectal cancer. *Lancet* **345**: 1257–1260

Inoue S, Nakao A, Kasai Y, Harada A, Nonami T and Takagi H (1995) Detection of hepatic micrometastasis in pancreatic adenocarcinoma patients by two-stage polymerase chain reaction/restriction fragment length polymorphism analysis. *Jpn J Cancer Res* **86**: 626–630

Kadomatsu K, Tomomura M and Muramatsu T (1988) cDNA cloning and sequencing of a new gene intensely expressed in early differentiation stages of embryonal carcinoma cells and in mid-gestation period of mouse embriogenesis. *Biochem Biophysis Res Commun* **151**: 1312–1318

Kadomatsu K, Hagihara M, Akhter S, Fan QW, Muramatsu H and Muramatsu T (1997) Midkine induces the transformation of NIH3T3 cells. *Br J Cancer* **75**: 354–359

Kaname T, Kadomatsu K, Aridome K, Yamashita S, Sakamoto K, Ogawa M, Muramatsu T and Yamamura K (1996) The expression of truncated MK in human tumours. *Biochem Biophysis Res Commun* **219**: 256–260

Kojima S, Inui T, Muramatsu H, Kimura T, Sakakibara S and Muramatsu T (1995*a*) Midkine is a heat and acid stable polypeptide capable of enhancing plasminogen activator activity and neurite outgrowth extension. *Biochem Biophys Res Commun* **216**: 574–581

Kojima S, Muramatsu H, Amanuma H and Muramatsu T (1995*b*) Midkine enhances fibrinolytic activity of bovine endothelial cells. *J Biol Chem* **270**: 9590–9596

Kojima S, Inui T, Kimura T, Sakakibara S, Muramatsu H, Amanuma H, Maruta H and Muramatsu T (1995*c*) Synthetic peptides derived from midkine enhances plasminogen activator in bovine aortic endothelial cells. *Biochem Biophys Res Commun* **206**: 468–473

© Cancer Research Campaign 1998

Matsuda Y, Talukder AH, Ishihara M, Hara S, Yoshida K, Muramatsu T and Kaneda N (1996) Limited proteolysis by chymotrypsin of midkine and inhibition by heparin binding, *Biochem Biophys Res Commun* **228**: 176–181

Merenmies J and Rauvala H (1990) Molecular cloning of the 18 kDa growth-associated protein of developing brain. *J Biol Chem* **265**: 6721–6724

Miyashiro I, Kaname T, Nakayama T, Nakanori S, Yagyu T, Monden T, Kikkawa N, Nishisho I, Muramatsu T, Monden M and Akiyama T (1996) Expression of truncated midkine in human colorectal cancers, *Cancer Lett* **106**: 287–291

Miyashiro I, Kaname T, Eisei S, Wakasugi E, Monden T, Takatsuka Y, Kikkawa N, Muramatsu T, Monden M and Akiyama T (1997) Midkine expression in human breast cancers: expression of truncated form. *Breast Cancer Res Treat* **43**: 1–6

Mori M, Mimori K, Inoue H, Barnard GF, Tsuji K, Nanbara S, Ueo H and Akiyoshi T (1995) Detection of cancer micrometastases in lymph nodes by reverse transcriptase-polymerase chain reaction. *Cancer Res* **55**: 3417–20

Muramatsu H and Muramatsu T (1991) Purification of recombinant midkine and examination of its biological activities: functional comparison of new heparin binding factors. *Biochem Biophys Res Commun* **177**: 652–658

Muramatsu H, Inui T, Kimura T, Sakakibara S, Song X, Murata H and Muramatsu T (1994) Localization of heparin-binding, neurite outgrowth and antigenic regions in midkine molecule, *Biochem Biophys Res Commun* **203**: 1131–1139

Muramatsu T (1993) Midkine (MK), the product of a retinoic acid responsive gene, and the pleiotrophin constitute a new protein family regulating growth and differentiation. *Int J Dev Biol* **37**: 183–188

Nakagawara A, Milbrandt J, Muramatsu T, Deuel TF, Zhao H, Cnaan A and Brodeur GM (1995) Differential expression of pleiotrophin and midkine in advanced neuroblastomas, *Cancer Res* **55**: 1792–1797

O'Brien T, Cranston D, Fuggle S, Bicknell R, Harris AL (1996) The angiogenic factor midkine is expressed in bladder cancer, overexpression correlates with a poor outcome in patients in invasive cancers. *Cancer Res* **56**: 2515–2518

Schulte AM, Lai S, Kurtz A, Czubayko F, Riegel AT and Wellstein A (1996) Human trophoblast and choriocarcinoma expression of the growth factor pleiotrophin attributable to germ-line insertion of an endogeneous retrovirus. *Proc Natl Acad Sci* **93**: 14759–14764

Tomomura M, Kadomatsu K, Matsubara S and Mutamatsu T (1990) A retinoic acid-responsive gene, MK, found in teratocarcinoma system. Heterogeneity of the transcript and the nature of the translation product. *J Biol Biochem* **265**: 10765–10770

Tsutsui J, Kadomatsu K, Matsubara S, Nakagawara A, Hamanoue M, Takao S, Shimazu H, Ohi Y and Muramatsu T (1993) A new family of heparin binding growth/differentiation factor: increased midkine expression in Wilms' tumor and other human carcinomas. *Cancer Res* **53**: 1281–1285

Tsutsui J, Uehara K, Kadomatsu K, Matsubara S and Muramatsu T (1991) A new family of heparin-binding factors: strong conservation of midkine (MK) sequences between the human and the mouse. *Biochem Biophys Res Commun* **176**: 792–797

© Cancer Research Campaign 1998